# Aragonite toothpaste for management of dental calculus: A double‐blinded randomized controlled clinical trial

**DOI:** 10.1002/cre2.559

**Published:** 2022-04-14

**Authors:** Ashwaq A. Al‐Hashedi, Nadia Dubreuil, Timothy Schwinghamer, Subad Dorzhiyeva, Lamyia Anweigi, Elham Emami, Faleh Tamimi

**Affiliations:** ^1^ Faculty of Dentistry McGill University Montreal Quebec Canada; ^2^ Cégep Garneau Quebec City Quebec Canada; ^3^ Agriculture and Agri‐Food Canada Lethbridge Alberta Canada; ^4^ Cégep Vanier Montreal Quebec Canada; ^5^ College of Dental Medicine, QU Health Qatar University Doha Qatar

**Keywords:** aragonite, dental calculus, oral hygiene, toothpaste

## Abstract

**Objectives:**

Aragonite from animal origin such as cuttlefish bone powder is an abrasive with hardness properties ideal for calculus removal. The purpose of this randomized controlled trial was to test the efficacy of a cuttlebone‐derived aragonite toothpaste in removing dental calculus.

**Materials and Methods:**

Eighty‐one patients who fulfilled the inclusion criteria were blindly and randomly assigned into two study groups. The intervention treatment group (*n* = 40) received cuttlebone toothpaste (Dr. D‐Tart) and the control group (*n* = 41) received an off‐the‐shelf commercial toothpaste (Crest). Evaluations were performed before and after scaling and polishing procedures done at 3 months in order to evaluate the toothpaste's ability to remove calculus and to prevent calculus formation. Calculus, stains, plaque, and gingival indices scores, and patient satisfaction surveys were compared at baseline (first visit), 3, and 9 months, using generalized linear models and Wald's *χ* ^2^ test.

**Results:**

At the end of the 3‐month period, the intervention group showed a 30% reduction in total calculus compared to the baseline score (*p* = .0006) and 45% less total calculus compared to the control group (*p* = .0001). Six months after scaling, the mean calculus score for Crest users was 42% higher than that for Dr. D‐Tart users (*p* = .0692). There was a significant improvement in the gingival health of cuttlebone toothpaste users at the observed intervals, and both kinds of toothpaste achieved comparable results in terms of plaque and stains removal.

**Conclusions:**

Aragonite toothpaste can remove calculus, prevent calculus formation, and improve gingival health. Patients are generally satisfied with the performance of the aragonite toothpaste. Clinical significance: Animal‐derived aragonite toothpaste (Dr. D‐Tart) shows promising efficacy in removing calculus, preventing calculus formation, and for the improvement of gingival health. Clinical trial ID: A08‐M35‐16B.

## 1 INTRODUCTION

Periodontal disease is amongst the most prevalent oral diseases worldwide and in North America (Canadian Health Measures Survey, 2010; P. Eke et al., 2012; P. I. Eke et al., 2018; Ismail et al., 1994; Tremblay et al., 2007). According to the 2007/2009 Canadian Health Measures Survey, 16% of Canadian adults and 24% of older adults, 60–79 years of age, were found to have moderate periodontal disease (Brown et al., 2000). Moreover, 11% of Canadian adults were found to have calculus scores in the highest range (Canadian Health Measures Survey, 2010). For the year 1999, estimates showed that the expenditure for periodontal treatments in the United States was about $14.3 billion (Tremblay et al., 2007). Periodontitis is mainly caused by calculus, the calcified form of dental plaque. Calculus carries several pathogenic microbes that cause periodontitis. If not treated, periodontal inflammation may cause progressive destruction of periodontal tissues (Najeeb et al., 2016). Dental calculus is a calcified mineralized plaque composed primarily of calcium phosphate salts covered by an unmineralized bacterial biofilm layer (Roberts‐Harry & Clerehugh, 2000). It is firmly attached to the tooth, filling the pits and irregularities of tooth surfaces (Jowett et al., 2013). Calculus can form both at the supragingival and subgingival levels. Subgingival calculus is more virulent and can only be removed by professional treatment; however, supragingival calculus is more accessible to cleaning tools and could be controlled by the patient. Supragingival calculus has been related to the presence of the more damaging subgingival calculus and to progressive periodontal disease (Jepsen et al., 2011; Nogueira Moreira et al., 2000). Thus, the removal of supragingival calculus can help improve the health of the periodontal tissues by reducing subgingival levels of pathogenic bacteria and reducing pocket depths and gingival inflammation and bleeding (Jepsen et al., 2011; Nogueira Moreira et al., 2000).

Dental calculus plays an important etiological role in the initiation and progression of periodontal diseases (Adriaens & Adriaens, 2004; Roberts‐Harry & Clerehugh, 2000), and the removal of it maintains adequate periodontal health (Lang et al., 2008). Currently, conventional tooth brushing is unable to remove calculus that is hard and resistant (Schiff et al., 2005). The most effective means to control dental calculus is to remove it mechanically by dentists and dental hygienists, mainly by scaling and polishing. This labor‐intensive procedure poses a burden to patients in terms of costs and access to dental professionals (Krause et al., 2007), and it can cause possible cementum and dentin substance loss. In this regard, several kinds of toothpaste have been developed to dissolve and soften the mineralized deposits using abrasive or chemical agents, such as crystallization inhibitors and mucinase. Toothpastes with strong abrasives are associated with a high risk of abrasive damage to tooth structures (Canadian Health Measures Survey, 2010), while toothpastes with anticalculus chemical agents are effective in reducing calculus deposition, but they do not remove formed calculus (Cvjetinovic et al., 2020; Jowett et al., 2013; Krause et al., 2007), and they affect supragingival calculus but not subgingival calculus (Jepsen et al., 2011).

Ideally, abrasives for calculus removal should be able to remove calculus without affecting dentin. This means that the hardness of the abrasive should be higher than that of calculus, but lower than dentin and cementum. The performance of abrasive particles such as those found on toothpaste also depends on particle size, morphology, charge, and toughness (Lee et al., 2010). For example, wear rates increase when the surface films are polished and the abrasives are oppositely charged (Sharath & Babu, 2004); in addition, for small abrasives, the wear rate increases proportionally with the increase in the abrasive particle size until it reaches the critical particle size (CPS). After reaching the CPS, the wear rate changes (Coronado & Sinatora, 2011).

Regarding particle morphology, particles with high attack angles tend to abrade by cutting through the surface, whereas particles that are less sharp would abrade by ploughing the surface (Coronado & Sinatora, 2011; Sevima & Eryurekb, 2006; Zum Gahr, 1987). Also, the properties of the surface play a role in its wear resistance. For example, low fracture toughness materials such as dental calculus wear by fragmentation; on the other hand, tougher materials such as dentin wear by ploughing and cutting mechanisms. In this sense, abrasives for calculus removal should be optimized for fragmentation wear rather than ploughing or cutting mechanisms (Gates & Gore, 1995).

The cuttlefish is a marine animal from the family cephalopods that has a unique inner mineralized tissue called the cuttlebone. This sophisticated buoyancy device is made of extensive superposed chambers that have a complex internal arrangement of calcified pillars made of the mineral calcium carbonate in its aragonite (orthorhombic) form and organic membranes made of a chitin–protein complex (Checa et al., 2015). Chitin is a modified polysaccharide with well‐known antibacterial and anti‐inflammatory activity that suppresses the production of inflammatory cytokines and favors fibroblast migration (Benhabilesa et al., 2012; Lim et al., 2015). The hierarchically organized aragonite is made of self‐organized diverse biomineral structures characterized by complex morphologies of hierarchically organized aragonite nanocrystals. With sharp angle forms that are ideal for abrasive particles design to achieve fragmentation and cutting wear (Čadež et al., 2017). For the reasons mentioned above, cuttlebone powder has been suggested as a potential useful abrasive for toothpastes for calculus removal; however, this has been never scientifically proven, and no clinical study has assessed this possibility.

Therefore, recently a new aragonite toothpaste made from cuttlebone has been developed specifically for calculus removal (Dr. D‐Tart toothpaste; Visionaturolab Inc., Terrebonne, QC, Canada). Our preliminary in vitro results showed that this toothpaste can selectively remove substantial amounts of calculus from tooth surfaces without damaging dentin (Kamath & Umesh Nayak, 2014). Building on this preclinical preliminary work, we have designed a randomized double‐blinded controlled clinical trial to assess the effect of cuttlebone toothpaste on dental calculus removal.

The aim of this study was to assess the efficacy of Dr. D‐Tart toothpaste in removing dental calculus compared to an off‐the‐shelf antitartar toothpaste (Crest® Complete Whitening Plus Scope, tartar control; Procter & Gamble, Cincinnati, OH, USA) (DIEYE & NDIME, 2015).

The working hypothesis was that there is a statistically significant difference between Dr. D‐Tart and control toothpaste in terms of dental calculus removal capacity. In addition, secondary outcomes such as dental stains and patient satisfaction were also assessed.

## 2 MATERIALS AND METHODS

### 2.1 Study design

The study was conducted as a double‐blinded randomized controlled parallel‐group trial. This study was approved by the human subjects' ethics board at McGill University, Montreal, Quebec, Canada (application A08‐M35‐16B) and was conducted in accordance with the Declaration of Helsinki 1975, as revised in 2013. The intervention group received Dr. D‐Tart toothpaste to remove calculus and stains on their teeth, while the control group received an off‐the‐shelf toothpaste (Crest Complete Whitening Plus Scope, tartar control; Procter & Gamble, Cincinnati, OH, USA). Besides aragonite, Dr. D‐Tart toothpaste ingredients also include water, humectants, sorbitol, thickening silica, surfactants, and stabilizers. The active ingredients in the Crest toothpaste include sorbitol, disodium pyrophosphate, and sodium lauryl sulfate. Crest toothpaste was chosen because its abrasiveness, sorbitol content, and pH are comparable to Dr. D‐Tart toothpaste, and therefore these characteristics were controlled in order to prevent unwanted interactions (Comité dentifrice of Collège François‐Xavier‐Garneau, 2007; Dieye & Ndime, 2015). The recruitment and data collection was done by a hygienist certified by the Quebec Federation of Dental Hygienists and the College of Dental Hygienists of Quebec. The recruitment started in April 2017 and lasted until March 2018. After a baseline assessment upon recruitment, each participant received appointments after 3, 6, and 9 months in order to evaluate the effectiveness of the toothpastes in removing calculus and preventing its formation.

### 2.2 Inclusion criteria

Participant's inclusion criteria in the study were as follows: (1) aged 18 years and over for both genders, to ensure the compliance with the study instructions; (2) systemically healthy; (3) participants had to have at least 20 sound natural teeth including and all lower anterior teeth, the main location of calculus build up; (4) participants had to have a history of previous calculus formation (at least 1.5 mm of calculus width) on the lingual surfaces of the mandibular anterior teeth after 6–9 months of receiving a professional prophylaxis treatment (from the patient file) in order to be able to evaluate the effect of the toothpaste; (5) participants had to agree to follow the study instructions and adhere to the allocated study arm for the study timeline.

### 2.3 Exclusion criteria

Subjects were excluded from participating in the study if they met these exclusion criteria: (1) any physical handicap, psychological, or health conditions that might jeopardize the ability of the patients to brush their teeth and/or attend the appointments; (2) use of antibiotics or anti‐inflammatory drugs within 1 month before the study, to avoid bias when assessing the gingival health; (3) regular use of chlorhexidine oral products, since these products can affect biofilm and calculus formation and introduce bias in our assessment of calculus removal by our toothpaste; (4) sensitivity to tartar‐control toothpastes; (5) presence of oral prostheses, dental implants, or fixed orthodontic appliances on teeth that will be included in the assessment since these devices can introduce bias to the study because they tend to increase the rate of plaque accumulation; (6) patients currently receiving dental treatment that would result in the removal of plaque or calculus and compromise our ability to measure calculus removed by the toothpastes; (7) patients unable to return for evaluations/study recalls, to avoid the significant loss of participants; (8) advanced periodontitis in the form of by having a Periodontal Screening and Recording (PSR) scale of 4; these patients have a high risk of tooth loss that could compromise calculus measurements during the study; (9) pregnancy, since it could prevent patients from complying with long evaluation sessions due to delivery and postpartum responsibilities; in addition, the gingival inflammation that usually occurs during pregnancy could introduce bias to the study.

Patients were recruited through advertisements on the radio, newspapers, social media, the Quebec Federation of Dental Hygienists, College of Dental Hygienists of Quebec, and the advertising board at Cégep Garneau and Université Laval. A website was made available for the public, in which potential participants were able to find the protocol, inclusion and exclusion criteria, and forms to complete for people who were interested to join. Participants interested in joining the study were asked to fill out the dental and medical history forms. Suitable participants who fulfilled the study criteria were contacted and given an opportunity to ask questions, discuss the study protocol with the dental hygienist and to take the Research Consent Form home for further consideration, and they were enrolled in the study after providing informed consent.

Randomization of participants was carried out off‐site by a statistician at the Faculty of Dentistry at McGill University. After screening and clinical examination, all participants enrolled in the study were randomly assigned to either the intervention (D‐Tart toothpaste) or control (Crest toothpaste) groups following a computer‐generated randomization list. The D‐Tart and control toothpastes were provided in an identical packaging, except for a coded identification number. The receptionist in the clinic was responsible for the coding process and giving the coded toothpastes to the participants. The coding for the toothpastes was withheld from the examiners until data analysis was completed.

### 2.4 Clinical procedures and evaluation

The primary outcome of the study was the amount of calculus removed by the toothpastes. The secondary outcomes were the amount of extrinsic tooth stains removed from the upper and lower incisors and reduction in gingival inflammation, and patient satisfaction with the performance of the toothpastes. All clinical parameters were evaluated by one calibrated examiner at baseline (first visit), 3, and 9 months.

At baseline sessions, the following clinical parameters were recorded as reported in the literature (Volpe et al., 1965):

1. The calculus accumulation on the lingual aspects of the six mandibular anterior teeth was assessed using the Volpe–Manhold Calculus Index score (Volpe et al., 1965).
2. The extrinsic stains on the labial and palatal/lingual surfaces of the upper and lower central and lateral incisors was assessed using the Shaw and Murray Stain Index (Shaw & Murray, 1977).
3. The plaque accumulated on the labial and lingual surfaces of teeth was measured using the Quigley–Hain Plaque Index (QHI) (Turesky et al., 1970).
4. The gingival health of the buccal and lingual marginal gingiva and interdental papillae of all teeth was assessed using the Modified Gingival Index (MGI) (Lobene et al., 1985).

After the initial evaluation, each participant was provided with the toothpaste and standard advice on tooth brushing (modified Stillman brushing technique), as well as a standardized oral hygiene kit that included an ultrasoft toothbrush (Gum 475 Microtip Copm Ultrasoft Comp Toothbrush, Sunstar Americas, Inc., Schaumburg, IL), end‐tuft toothbrush (Gum 308 End Tuft Tooth Brush, Sunstar Americas, Inc., Schaumburg, IL), a box of small interproximal brushes (Gum Proxabrush Go‐Betweens 414, Sunstar Americas, Inc., Schaumburg, IL), a box of large interproximal brushes (Gum Proxabrush Go‐Betweens 614, Sunstar Americas, Inc., Schaumburg, IL), and dental floss (Gum ButlerWeave waxed, Sunstar Americas, Inc., Schaumburg, IL). Each participant was trained to use the modified Stillman brushing technique and the following instructions were given:

- Brushing teeth: start brushing your lower teeth and then the upper teeth for 3 min, twice a day.
- Using toothpaste: use the size of a small pea for brushing, spit, and do not rinse.
- Using dental floss: with fingers or with dental floss holder: once a day.
- Using small brushes to clean the gaps between teeth (if necessary): once a day.
- Cleaning oral mucosa each time you brush.
- Mark on a customized calendar each time you brush your teeth with the allocated toothpaste.
- Do not use mouthwashes or other toothpastes for the duration of the study.
- Bring the toothpaste tubes and toothbrushes for assessment at each evaluation session.

Comparisons to baseline measurements and monitoring of the effect of the toothpastes before scaling during the first 3 months of the trial allowed assessment of their ability to remove dental calculus and manage gingivitis and stains and plaque, as most of the patients during this period had substantial calculus build‐ups. At 3 months, the participants were re‐examined, and the same data was recorded in order to evaluate the effectiveness of the toothpastes in removing the plaque, calculus, and stains, and maintaining gingival health.

At the same appointment, the participants were provided with a standard dental cleaning; thorough scale using ultrasonic and hand instruments followed by polishing with prophylaxis paste (fine bubble gum; Sunstar Butler, Guelph, ON, Canada) and topical application of neutral fluoride. Then, they were asked to continue using the same toothpaste and brushing technique/instructions that were described above. All the examinations were repeated for the participants at 6 and 9 months in order to evaluate the effectiveness of the toothpastes in preventing calculus formation. At the end of the trial (9‐month visit), the participants were offered a scale and polish free of charge as an incentive.

### 2.5 Patient satisfaction survey

A survey was performed at each one of the follow‐up appointments (months 3, 6, and 9) to evaluate the participants' satisfaction with the toothpastes; the survey consisted of seven questions that were answered using a visual analog scale (VAS) that ranged from highly satisfied to not satisfied at all, as follows:

Q1 How satisfied you are with the cleaning effect of the toothpaste?
Q2 How satisfied you are with the toothpaste taste?
Q3 How satisfied you are with the toothpaste texture?
Q4 How satisfied you are with the toothpaste consistence?
Q5 How satisfied you are with the toothpaste stickiness?
Q6 How satisfied you are with the toothpaste quality?
Q7 How is your overall satisfaction with the current toothpaste compared to other brands?

In addition, the survey had two additional open‐ended questions to register either “negative” or “positive” comments.

### 2.6 Statistical analysis

#### 2.6.1 Reliability of measurements

Two experienced dental hygienists were trained and calibrated on the scoring systems of all indices used. The intraexaminer data were analyzed by calculation of Cohen's *κ* test, which showed excellent agreement and significantly reliable results for all clinical measurements (*κ* = 0.92–0.96).

#### 2.6.2 Sample size calculation

According to previous clinical trials studies comparing toothpastes for calculus removal, anticalculus agents can reduce the amount of calculus by 30%–50% (Krause et al., 2007). A sample size of 40 per group was required to detect a clinically relevant difference (4%) between test and control toothpastes that achieved a study power of 80% at a significant level of .05 (Canadian Health Measures Survey, 2010; Jowett et al., 2013; Lobene et al., 1985; Najeeb et al., 2016). To allow a 10% dropout, the final sample of 90 participants was recruited (Power and Sample Size Calculations software, version 3.0, Vanderbilt University, Nashville, TN, using a two‐sided independent‐sample *t *test).

The normality of data distribution was initially tested. Demographic variables at baseline were compared using Fisher's exact test and Pearson's *χ* ^2^ test. Calculus, stain, QHI, and MGI scores were compared at 3, 6, and 9 months, using a generalized linear mixed models analysis with Wald's *χ* ^2^ test (Brown et al., 2000). Comparison between groups regarding VAS scores for the satisfaction survey was done with Student's *t *test and data were presented as mean and standard deviations; on the other hand, analyses for complaints and compliments was done using Pearson's *χ* ^2^ and data were presented as odds ratios. The *p *values were considered statistically significant if less than .05. There were no changes made in the study design after institutional review board approval and trial registration; the study proceeded in accordance with the protocol after the approval.

## 3 RESULTS

**Figure 1:**
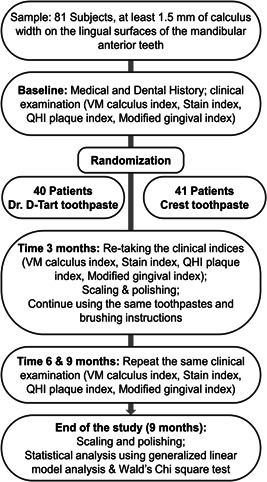
Flowchart showing the study design. QHI, Quigley–Hain Plaque Index; VM, Volpe–Manhold

Eighty‐three participants (24 males and 56 females) aged 18–69 years (mean: 43.8 ± 15 years) were enrolled in this study. Forty participants in the experimental group and 41 participants in the control group were evaluated and included in the analyses at baseline (time 0) and on the follow‐up time points at months 3, 6, and 9 as detailed in Figure 1.

### 3.1 Baseline data

**Figure 2:**
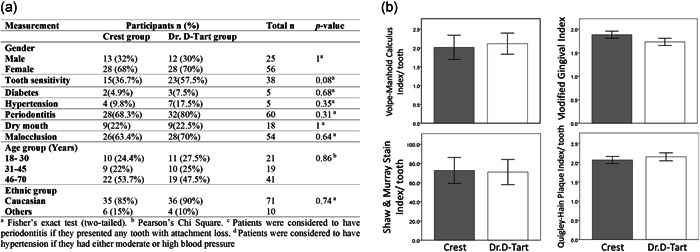
(a) Demographic data of two study groups at baseline, showing gender, age, and ethnic group. Values are expressed as the number and percent of subjects in each group. (b) Bar charts illustrating the comparison of clinical indices scores in the study groups after assigning them to the Crest and Dr. D‐Tart toothpastes (baseline). Scores are expressed as means ± standard errors of the means. No significant differences between study groups were observed

The homogeneity of demographic statistics and clinical parameters between study groups was evaluated at baseline (Figure 2). There was no statistically significant difference between the study groups in terms of demographic and clinical data (*p* > .05), indicating that the groups were well‐balanced and homogenous.

### 3.2 Calculus removal (Figure 3)

The result of comparisons of the baseline measurements and monitoring of the effect of the toothpastes indicated a 15% increase in calculus buildup with the use of Crest toothpaste (*p* = .03), while the aragonite toothpaste resulted in a 30% calculus reduction (*p* = .0006). At the end of this initial 3‐month monitoring period, the calculus buildup for the Crest group was 45% greater than that in the Dr. D‐Tart group (*p* = .0001). The MGI score decreased significantly by 30% among aragonite toothpaste users (*p* < .0001) and by 18% in the Crest group (*p* < .0001), indicating the statistically significant improvement of the gingival health of both aragonite and Crest users. The MGI scores of aragonite toothpaste users were 22% lower than the Crest users (*p* = .0059).

**Figure 3:**
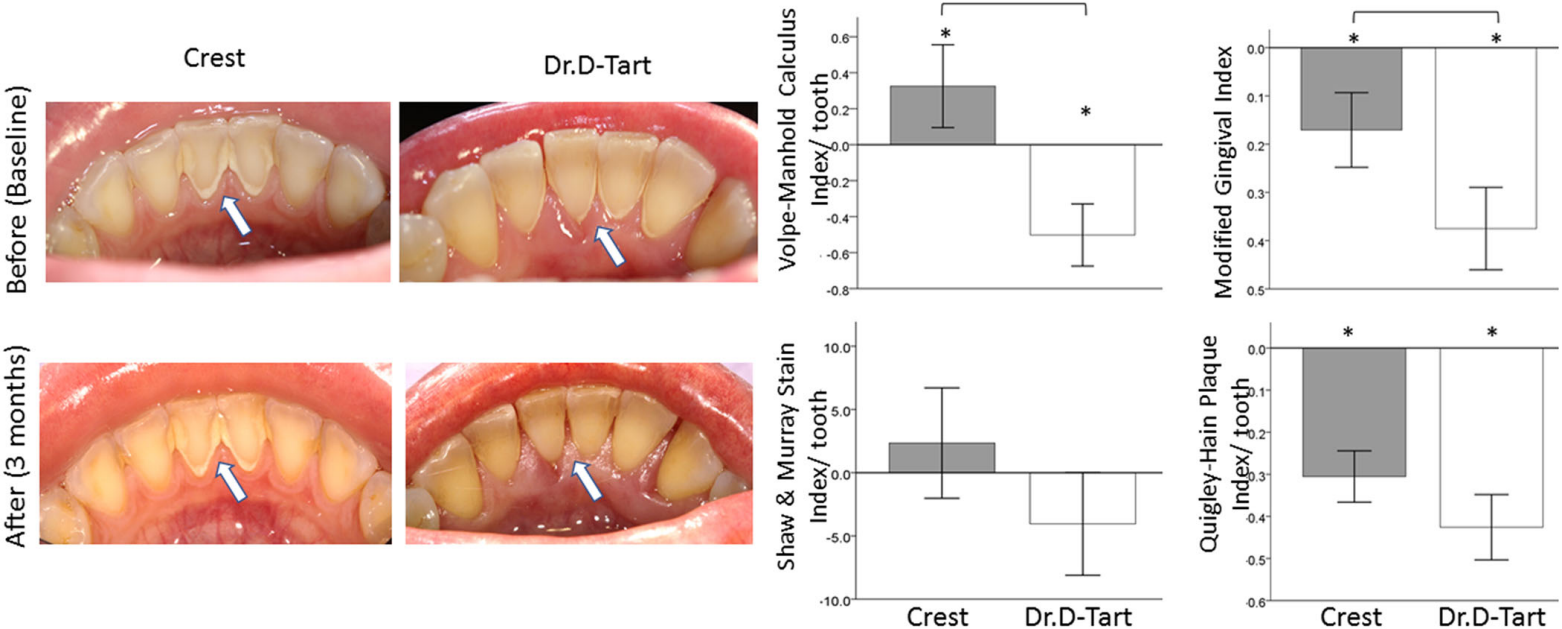
Photographs showing the labial/lingual aspects of lower anterior teeth and bar charts comparing the changes in clinical indices scores in the study groups (Crest and Dr. D‐Tart toothpastes) after 3 months of toothpaste usage. Scores are expressed as means ± standard errors of the means. *Significantly different from baseline score, before using the toothpaste (*p* < .05). Brackets indicate significant differences between groups (*p* < .05).

The plaque index was statistically significantly lower than the baseline observation, for both study groups (*p* < .0001) with no significant difference in the mean plaque score between the two study groups (*p* = .78). The mean stain scores did not change to a statistically significant degree after 3 months, in comparison to the baseline (time 0) for both study groups (*p* < .05), indicating that the toothpastes were comparable in removing stains during the first 3 months of the study (*p* = .59). PSR scale was lower than the baseline time of observation for both study groups, although there was no significant difference in the mean PSR between the two study groups (*p* = .31).

### 3.3 Prevention of calculus buildup, gingival inflammation stains, and plaque accumulation (Figure 4)

At the 6‐month appointment (3 months following initial scaling), the study groups were characterized by having statistically significantly less calculus accumulation than that at the baseline time point. At the 9‐month visit (6 months after scaling), the aragonite toothpaste group showed 42% less calculus than the Crest group, but the differences were not statistically significant (*p* = .0692), and the mean score of MGI for the aragonite group was 40% lower than that for the Crest group, and the differences were significant (*p* < .0001).

**Figure 4:**
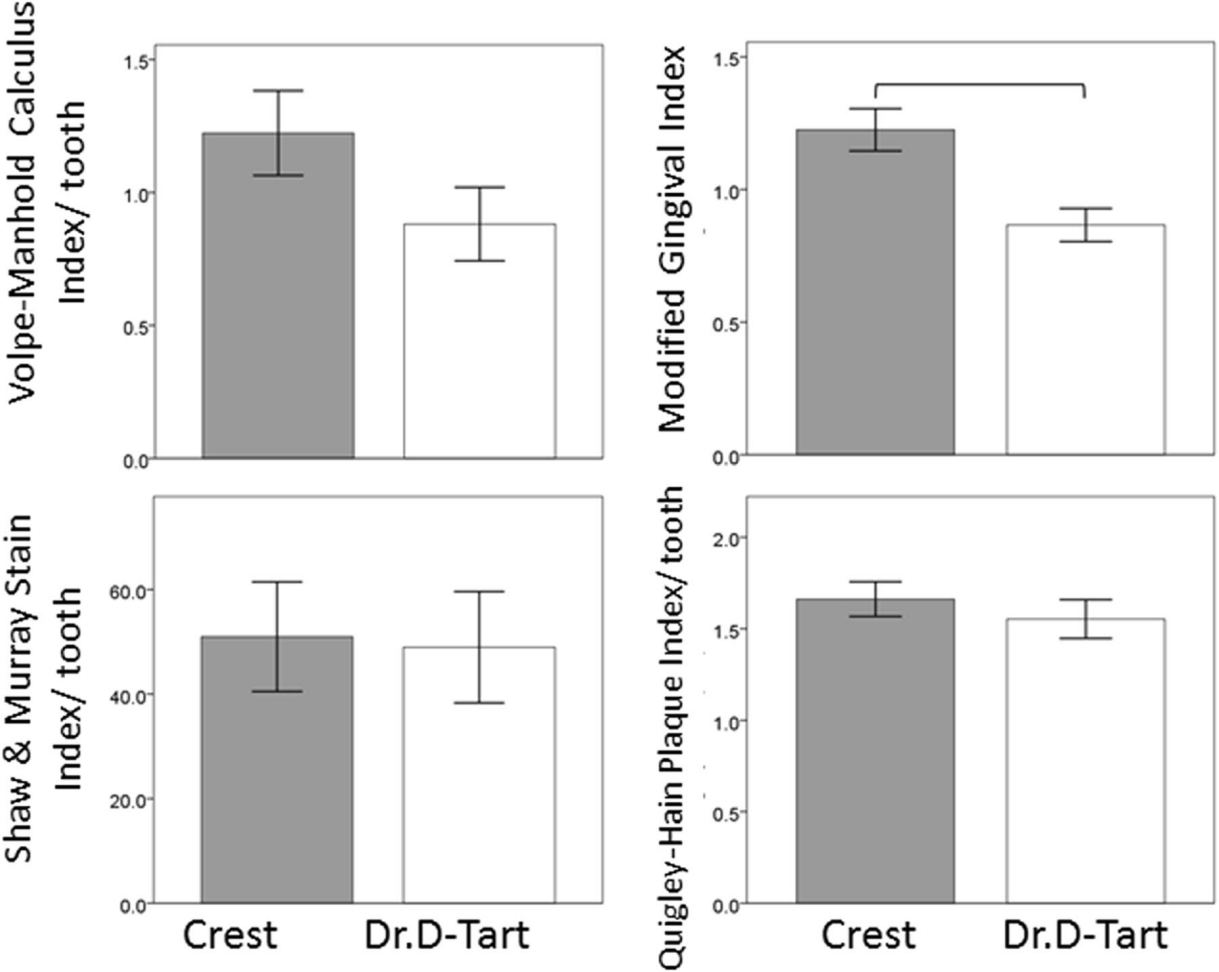
Bar charts comparing the clinical indices scores in the study groups (Crest and Dr. D‐Tart toothpastes) 6 months after scaling (9‐month time point). Scores are expressed as means ± standard errors of the means. The brackets indicate significant differences between the Crest and Dr. D‐Tart groups (*p* < .05)

Also, at the 9‐month visit (6 months after scaling), the mean stain and plaque index scores were statistically significantly lower than that at the baseline for both study groups (*p* < .0001), and there were no statistically significant differences between the two study groups. This indicates that the aragonite toothpaste could be comparable to Crest toothpaste in terms of prevention of dental stains (*p* = .91) and plaque accumulation (*p* = .3).

### 3.4 Patient's safety and satisfaction

**Figure 5:**
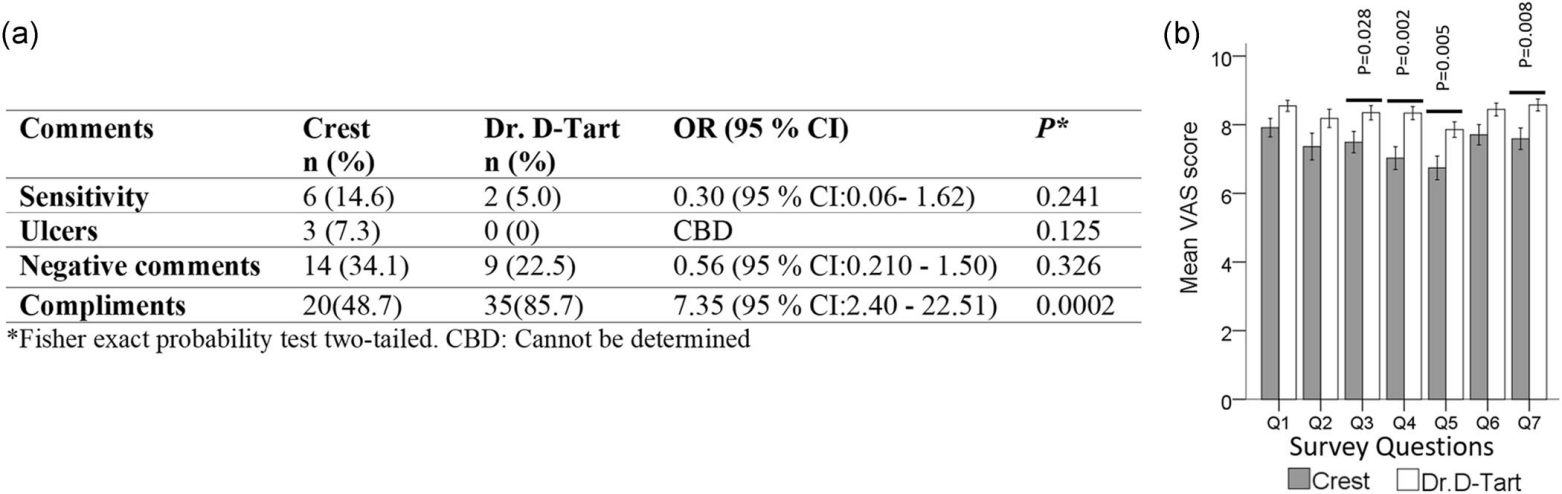
(a) Analysis of comments by patients and (b) results of patient satisfaction survey at month 9. Brackets indicate significant differences between groups at each time interval (*p* < .05). Statistical analysis was done with Student's *t *test. CAB, cannot be determined; CI, confidence interval; VAS, visual analog scale

The participants in the study groups experienced no adverse events during the study and the toothpastes were well tolerated. A total of 14 patients reported a negative comment in the control group compared to nine patients in the aragonite group: six patients reported tooth sensitivity in the control group compared to two patients in the aragonite group, and three patients reported an incident of an oral ulcer in the control group compared to none in the aragonite group. However, the differences between the control and experimental group in terms of negative events and complaints were not statistically significant. On the other hand, patients in the aragonite group reported a statistically significantly higher number of compliments (Figure 5a).

**Figure 6:**
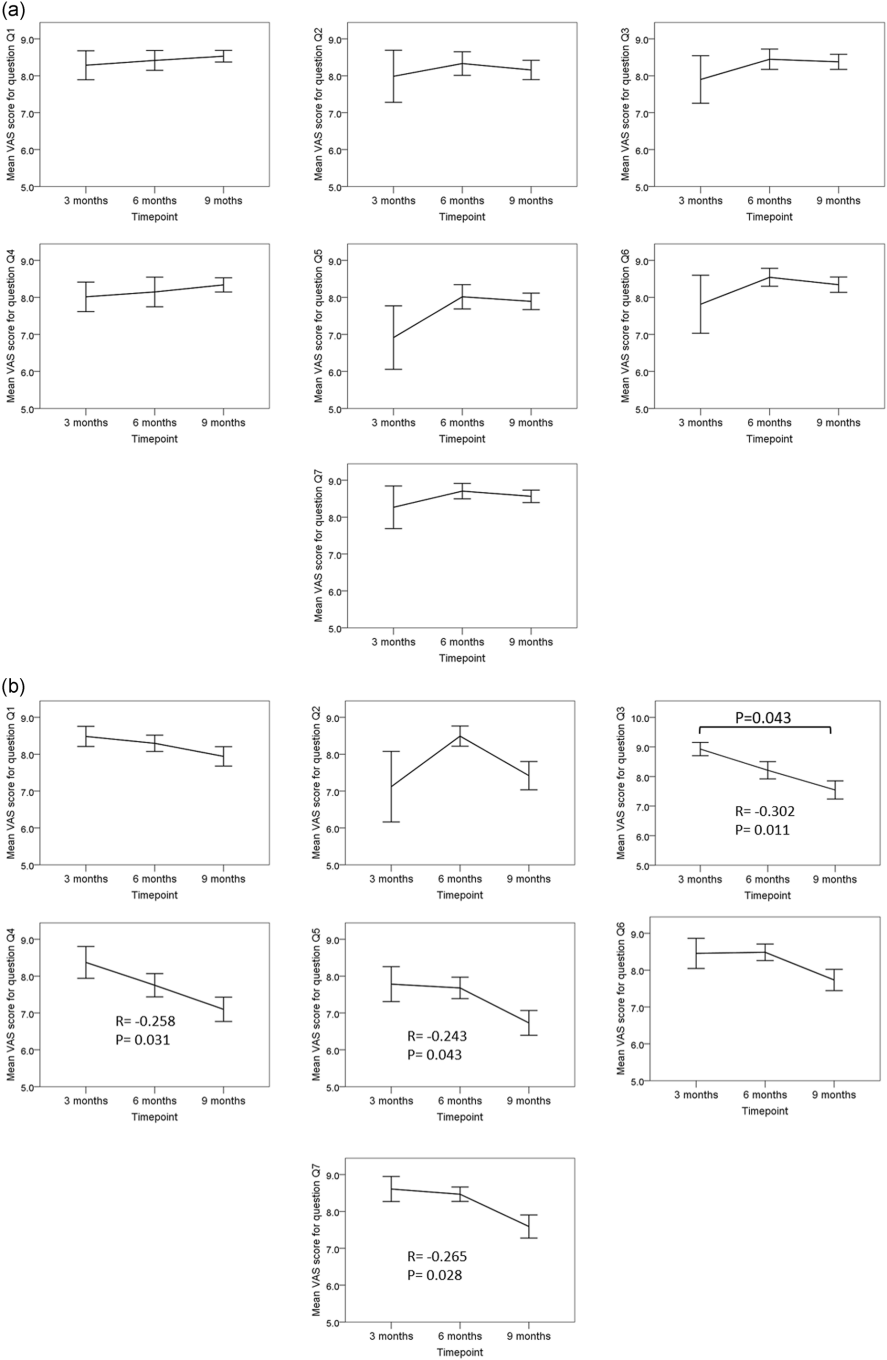
(a) Results of the survey on Dr. D‐Tart toothpaste for the different questions at different timepoints. No significant correlations between the question scores and the timepoints and no significant differences between time points were found. (b) Results of the survey on Crest toothpaste for the different questions at different timepoints. Bracket indicates statistically significant difference (*p* < .05) between timepoints. Statistical analysis was done with one‐way analysis of variance with post hoc Bonferroni test. Significant Pearson's correlation coefficients are presented as insets in the graphs

The patient satisfaction VAS survey revealed no statistically significant difference between groups after 3 and 6 months of using the toothpastes; however, after 9 months patients using the aragonite toothpaste registered significantly higher satisfaction scores in four categories (Figure 5b): texture (Q3), consistence (Q4), stickiness (Q5), and overall satisfaction (Q7). Also, there was a slight increase in the satisfaction scores among patients treated with aragonite toothpaste and a slight decrease in the satisfaction scores in the crest group (Figure 6) that was statistically significant for questions on texture (Q3), consistence (Q4), stickiness (Q5), and overall satisfaction (Q7).

## 4 DISCUSSION

To our knowledge, this is the first RCT to assess the performance of a toothpaste using cuttlebone powder as an abrasive. Our results indicated that the toothpaste cuttlebone aragonite (Dr. D‐Tart) was effective in calculus removal, prevention of calculus formation, and for the improvement of gingival health. The patient satisfaction survey data indicated that patients treated with the aragonite toothpaste were statistically significantly more satisfied and that they suffered no major side effects. We discuss these results in detail below.

### 4.1 Calculus removal

During the first 3 months of our study, no scaling was performed. Therefore, calculus reduction during this period was indicative of the calculus removal capabilities of the toothpastes tested. In our control group, there was a slight increase in the amount of calculus, which indicates that Crest toothpaste did not remove calculus. This is consistent with the literature on toothpaste for calculus control, reporting that anti‐alculus toothpastes do not remove calculus that has already formed, but rather help prevent the formation of additional calculus (Dupont & Plummer, 1990; White, 1997). On the other hand, patients who used the aragonite toothpaste were characterized by a lower level of calculus accumulation. This result indicates that cuttlebone‐derived aragonite toothpaste removes calculus. This is probably related to the unique hardness and specific surface area of the cuttlefish bone powder abrasives (North et al., 2017). Other anticalculus toothpastes relied on chemical agents for crystal growth inhibition and hard silica abrasives for stain removal. This has limitations because crystal‐growth inhibitors can prevent calculus buildup, but they do not contribute to calculus removal, while silica abrasives are harder than the tooth enamel and dentin, which limits the amount that could be used in toothpaste to achieve cleaning efficiency without causing tooth damage (Fairbrother & Heasman, 2000; Franzo et al., 2010; Joiner et al., 2008). On the other hand, cuttlebone powder is an abrasive that is softer than tooth enamel and dentin and it is characterized by a relatively high surface area.

### 4.2 Prevention of calculus formation

After the first scaling, the patients treated with the aragonite toothpaste exhibited low levels of calculus over a prolonged period compared to the patients treated with the Crest toothpaste who showed a progressive increase in calculus buildup. Over the 6‐month period that followed the scaling session, the mean calculus score for Crest users was 42% higher than that for the users of the aragonite toothpaste.

Prevention of calculus formation is a feature described in several commercialized toothpastes that are achieved by two types of active ingredients: chemical products that prevent crystal growth and mechanical abrasives (Fairbrother & Heasman, 2000). Among the chemical crystal growth inhibitors used in toothpastes for the prevention of calculus formation, zinc citrate trihydrate reduces calculus formation by 30% over 13 weeks, sodium hexametaphosphate inhibits calculus compared to triclosan/copolymer dentifrice after 6 months, and pyrophosphate (3.3%) reduces calculus accumulation by 32.3% after 6 months (Grases et al., 2009; Liu et al., 2002; Segreto et al., 1998). Also, toothpastes with stannous fluoride (0.454%) and sodium hexametaphosphate (13%) reduce calculus buildup by 56% at 6 months compared to triclosan/copolymer toothpastes (Schiff et al., 2005). Sodium polyaspartate and arginine have no significant advantage regarding calculus deposition (Jowett et al., 2013).

Regarding calculus control using abrasive agents, toothpastes with high cleaning grade silica particles achieve a statistically significant 34% reduction in mean Volpe–Manhold Calculus Index score as compared to a negative control dentifrice after 8 weeks (Sowinski et al., 2002).

In this context, the ability of the aragonite toothpaste assessed in this study is on the upper end of the reported literature on toothpaste design for the prevention of calculus buildup.

### 4.3 Gingival inflammation

Although some toothpastes, such as those containing triclosan, have been shown to be effective in improving gingivitis and periodontal health (Davies et al., 2010). So far, neither a therapeutic benefit (in terms of less gingivitis or less caries) nor a societal benefit (in terms of less treatment demand) has been demonstrated as a result of the anticalculus and whitening effects of toothpastes (Van Loveren & Duckworth, 2013). Thus, one unexpected finding in this study was that there was a significant improvement in the gingival health of aragonite toothpaste users compared to the Crest group. This is very relevant because gingival inflammation is a well‐known precursor for periodontal disease and reducing gingivitis could help reduce the risk of developing periodontal disease.

It could be assumed that the effect of cuttlebone aragonite toothpaste on gingival inflammation could be related to the reduction observed in calculus buildup. The presence of calculus is associated with gingival inflammation, and it is considered a secondary etiological factor for gingival inflammation. Indeed, toothpastes with no effect on calculus tend to have limited or no effect on gingivitis (Grases et al., 2009). Another potential reason behind the anti‐inflammatory effect of cuttelbone toothpaste could be its high content in sorbitol (10%), which has an anti‐inflammatory effect on gingival tissues (Saheer et al., 2019; Soeteman et al., 2018), although the crest toothpaste also had sorbitol as the active ingredient.

### 4.4 Stain removal

Regarding stain removal, both toothpastes were comparable in preventing tooth staining, as there were no significant differences between the dental stain indices of both groups.

Toothpastes able to remove extrinsic stains, such as the Crest toothpaste used in our study, are often referred to as whitening toothpastes (Hara & Turssi, 2017). These toothpastes usually contain chemical agents or abrasives that increase their ability to remove extrinsic stains and/or prevent their formation. Some examples of such chemicals include, for example, peroxide, enzymes, citrate, triclosan, baking soda, pyrophosphate and hexametaphosphate, and calcium carbonate (Hara & Turssi, 2017; Saheer et al., 2019; Soeteman et al., 2018; Van Loveren & Duckworth, 2013). In this context, it could be speculated that the aragonite present in the experimental toothpaste we tested had a whitening effect defined by its ability to remove stains, and this whitening effect was expected because the antistain whitening effects of toothpastes are to some extent based on the same compounds that achieve anticalculus effects (Van Loveren & Duckworth, 2013).

### 4.5 Patient satisfaction

One major concern with toothpastes designed for calculus removal is that their abrasiveness could cause side effects such as sensitivity or ulcers. Adverse reactions to toothpastes are rare but should be considered in unexplained skin or respiratory allergies and gingival or lip lesions (Davies et al., 2010).

Interestingly, the patients treated with the aragonite toothpaste reported fewer incidents of sensitivity or ulcers than the patients using the control toothpaste, which seems to indicate that aragonite toothpaste does not cause any clinically relevant side effects.

### 4.6 Strengths, limitations, and future directions

The main strength of this study is the randomization of the patients, which is confirmed by the similar baseline characteristics in both study groups. Another very important strength is the blinding at multiple levels; the patients were blinded to the type of treatment they received, the hygienist performing the measurements and the scaling were also blinded to group allocation. Another strength is the prolonged follow‐up that allowed us to assess the effect of the toothpastes long after a scaling intervention. Moreover, the standardization of the hygiene instructions for all patients was also a strength. All patients used the same oral hygiene kit, following the same oral hygiene instructions.

However, this study has some limitations that motivate future research. One of such limitations was the variability in calculus buildup across patients; anecdotally, it was observed that some patients exhibited very large and fast calculus buildups, while others exhibited less. This could be explained by specific conditions in each patient. One example is the reduced salivary flow observed in some patients that could have increased the speed of calculus build‐up. In addition, dietary or genetic factors were not considered in this study and that can be studied in the future. Another limitation is not standardizing patient's periodontal disease, including patients with health problems since their medications could also affect calculus formation. Nonetheless, to address these limitations, the individual patient was included as a random factor in the generalized linear mixed models.

In addition, even though the aragonite dentifrice was effective in managing dental calculus, the potential health benefits of the prevention of dental caries are doubtful since it does not contain fluoride. Future research and development would be needed to add fluoride to the cuttlebone‐derived aragonite toothpaste to address this issue.

In this study the aragonite toothpaste was compared only to single commercial toothpaste (Crest complete). The control toothpaste was selected because it has been specifically designed for tartar control. However, future studies would be needed to compare aragonite toothpastes with other types of toothpastes, including those that are not designed for tartar control in order to further test its effectiveness in calculus removal.

We believe these findings are internally valid as the study design minimized bias by double‐blinding and random distribution of confounders to treatment and control groups. However, external validity should be interpreted with caution due to the several and strict inclusion and exclusion criteria of this study.

## 5 CONCLUSION

Animal‐derived aragonite toothpaste could effectively remove calculus, prevent calculus formation, and improve gingival health. Moreover, participants are generally satisfied with the performance of animal aragonite toothpaste.

## AUTHOR CONTRIBUTIONS

Ashwaq A. Al‐Hashedi, Elham Emami, and Faleh Tamimi have made substantial contributions to the conception and design of the study. Ashwaq A. Al‐Hashedi, Nadia Dubreuil, Timothy Schwinghamer, and Subad Dorzhiyeva have been involved in data collection and data analysis. Ashwaq A. Al‐Hashedi, Nadia Dubreuil, Timothy Schwinghamer, Subad Dorzhiyeva, and Lamyia Anweigi have been involved in data interpretation. Ashwaq A. Al‐Hashedi, Elham Emami, Lamyia Anweigi, and Faleh Tamimi have been involved in drafting the manuscript. All authors have been involved in revising the manuscript critically and have given final approval of the version to be published.

## CONFLICTS OF INTEREST

Dr. Faleh Tamimi has aquired shares in the company that developed the aragonite toothpaste described in this study, Visionaturalab Inc. Also, this study was financially supported by Visionaturalab Inc., which is the company that developed the aragnoite toothpaste described in the study.

## Data Availability

The data that support the findings of this study are available on request from the corresponding author. The data are not publicly available due to privacy or ethical restrictions.
