# Aragonite toothpaste for management of dental calculus: A double‐blinded randomized controlled clinical trial

**DOI:** 10.1002/cre2.614

**Published:** 2022-06-29

Corrections:

Statistical analysis

Sample size calculation: (page 5, paragraph 1, line 8 & 9)

Author have stated about 10% dropout in the study and final recruitment of sample size 90 is not matching with the text elsewhere.

Results (page 5. Paragraph 1, lines 1&2)

The author have stated in the results that 83 participants were included in the study. Whereas, in the results the sum total (24 males and 56 females) does not add up to 83

